# Ethical sensitivity and compassion in home care: Leaders’ views

**DOI:** 10.1177/09697330221122965

**Published:** 2022-10-14

**Authors:** Heidi Blomqvist, Elisabeth Bergdahl, Jessica Hemberg

**Affiliations:** Faculty of Education and Welfare Studies, Department of Caring Sciences, 1040 Åbo Akademi University, Finland; School of Health Sciences, Örebro University, Sweden; Faculty of Education and Welfare Studies, Department of Caring Sciences, 1040 Åbo Akademi University, Finland

**Keywords:** Caring, compassion, ethical sensitivity, home-based care, home care leaders

## Abstract

**Background:**

With an increasing older population, the pressure on home care resources is growing, which makes it important to ensure the maintenance of quality care. It is known that compassion and ethical sensitivity can improve the quality of care, but little is known about care leaders’ perceptions on ethical sensitivity and compassion in home care and how it is associated with staff competence and thus quality of care.

**Aim:**

The aim of the study was to explore home care leaders’ perceptions of ethical sensitivity and compassion associated with care quality in home care.

**Research design, participants, and research context:**

A hermeneutical approach with a qualitative explorative design was used. The data consists of texts from 10 in-depth interviews with home care leaders. Content analysis was used as a method.

**Ethical considerations:**

The study was conducted following the ethical guidelines of the Declaration of Helsinki and the Finnish Advisory Board of Research Ethics. Research ethics permission was applied for from a Research Ethics Board.

**Findings:**

One overall theme and four subthemes were found. The overall theme was: “Compassion provides deeper meaning and ethical sensitivity provides means for knowing how to act”.

**Discussion:**

If nurses fail to be sensitive and compassionate with patients, good and high qualitative home care cannot be achieved. Ethical sensitivity and compassion can be seen as resources in home care but the organization and the care leaders need to provide the support for these to develop.

**Conclusion:**

This study provides an understanding of the meaning of ethical sensitivity and compassion as sources of strength and their link to quality of care in a home care context. Further studies could focus on how to build compassion and ethical sensitivity into home-based care and how to ensure adequate support for healthcare professionals’ compassion and ethical sensitivity.

## Introduction

While the need for home care is increasing globally, research shows that several countries cannot fully meet patients’ care needs.^1^ Internationally, healthcare is governed by human rights, healthcare legislation, and ethical guidelines.^2,3^ According to The National Advisory Board on Social Welfare and Health Care Ethics ETENE, older people should have the right to good qualitative care that respects the individual’s integrity, needs, and right to self-determination.^4^ Ethics as a core value in healthcare emphasizes professional behavior tied to care ethics and values, and care quality is determined not only by what or which care is given but also by how the care is given.^5^ Home care personnel have been singled out as one of the factors that can give older people meaningfulness in home care,^6^ and it is suggested that the performance of care has a bearing on care quality: as closely related to an organization’s ethical culture this puts home care leaders in a significant role because their impact on other healthcare professionals can affect the caregivers’ being and caring.^7^ Consequently, care leaders in an organization are in a strong position to influence care quality.^8^ Compassion as a basis for care ethics guides caregivers and care organizations, and Fotaki^9^ argues that it would be important to recognize on the organizational level the importance of compassion in caring because compassion otherwise risks being stifled by the environment.^10^ This study aims to investigate how ethical sensitivity and compassion are linked to staff competence and thus care quality in home care according to the perceptions of home care leaders.

### Background

Compassion as a concept comes from the Greek *sympatheia* meaning compassion, with the Latin equivalent being *compassion*.^11^ *Compassion* means a capacity to call for compassionate responses in others.^12^ *Compassion* is one of the five values required of professional nurses by the International Council of Nurses (ICN)^13^ and can be seen as the basis for the ethical codes of care.^3,9,14^ When it comes to good care quality, van der Cingel^15^ mentions compassion as the crucial link between good evidence-based care and the caring relationship that is created between caregiver and patient, and where the former performs professional care based on knowledge that is formed by intuition. Compassion can be described as the deep awareness of another human being’s suffering that creates an emotional touch that entails an action or motivation to help.^16,17^ Compassion is born when the caregiver encounters the other’s vulnerability and sees the suffering,^12,18^ which implies a deep awareness of the other’s suffering,^19^ and it is expressed through genuine involvement in the caring encounter and through the caregiver’s actions to alleviate this suffering.^20^ Compassion is expressed when human contact, reciprocity, and belonging come together.^20^ If suffering human beings are not seen in their suffering, they may perceive that their credibility is being questioned^19^ and, therefore, compassion should be allowed space in caring. The caregiver’s compassion becomes confirmation in the form of eye contact, touch, or words that show that the human being is seen.^19^ Consequently, showing compassion becomes a way of expressing reverence and respect for the human being. Compassion is described as *the heart*, or *the fundamental core of caring*.^20^ When compassion is present in caring, suffering can be alleviated and client health can be enhanced.^20^ The presence of compassion also improves care quality.^21^ Compassion is even described with concepts such as *kindness* and *security* and includes having an understanding of the other’s suffering.^16^ Furthermore, compassion is defined using concepts such as presence, time, and dignity^22^ and by nurses acting on the basis of their inner ethos, heart of goodness, and love.^20^ Compassion is moreover described as paying attention to patients’ interests and needs^15^ and caringly taking the time to communicate and create trust in the caring relationship through conversations.^23,18^ The presence of compassion in the care of older people is of great importance because older people are often vulnerable as a result of physical, cognitive, emotional, or social factors, which can indicate suffering.^24^ Compassion is not just something caregivers do, are, or feel, but it is about belonging to another person where both have a mutual commitment and where caregivers can acknowledge both themselves and the vulnerability and dignity of others.^25^ Hemberg and Wiklund Gustin^20^ describe this as being in a loving communion where nurses share their own vulnerability by, for example, being touched by the other’s suffering, which can encourage patients to show their own vulnerability. That the nurse is available to the patient in an honest and humble way in this loving communion means a belonging where the nurse and patient encounter each other as human beings and are present in the moment together as an expression of compassion that affirms the patient’s dignity.^20^

A suffering human being needs to be given time and space to suffer.^26,27^ Ethical sensitivity concerns recognizing and interpreting the ethical dimension of a care situation as the first aspect of decision-making in professional practice.^28^ To be treated and confirmed in one’s suffering can mean that dignity is maintained in the human being.^26,27^ *Ethical sensitivity* can be described as the caregiver’s ability to recognize an ethical dilemma or ethical aspects of a situation and a *sine qua non* for decision-making in healthcare practice.^29,30,31^ Ethical sensitivity implies the emotional ability to discern ethical tensions and is a prerequisite for other ethical components, for example, reflection, behavior, decision-making, and action.^32^ Ethical sensitivity is the component that starts from the caregiver’s being and entails the making: it is based on the caregiver’s attitudes, character traits and values, and leads to deeds.^32^ As a concept, ethical sensitivity can be considered part of the caregiver’s professional competence^33^ and ethical decision-making, where such awareness ensures the best interests of the patient through recognition of the patient and the patient’s needs, in line with the ethics of care and the wishes of the unique individual.^31^ The caregiver’s education and work experience influence his/her ethical sensitivity,^29,30^ and the caregiver’s ethical sensitivity and life experience in turn facilitate his/her moral development and actions.^33^ Ethical sensitivity also includes the caregiver’s ability to interpret verbal and/or nonverbal behaviors in order to best identify the patient’s needs.^29^ Hemberg and Bergdahl^34^ argue that ethical sensitivity and responsiveness through co-creation in palliative home care allows the caregiver to balance the care measures in the moment and respectfully take a step back to make room for the client’s expressed and/or unspoken or hidden (not expressly articulated) needs and desires. By imagining themselves in the client’s situation, caregivers can better understand the client’s physical and emotional needs, and through ethical sensitivity a mutual understanding can be created in the caring relationship.^33^ In a caring encounter where the caregiver demonstrates ethical sensitivity, the suffering person is given the time and space needed in that particular moment.^6^ The caregiver’s ethical sensitivity can have a positive impact on care quality^29,35^ as well as reduce the client’s stress.^30^

According to Huang et al.,^30^ there is a correlation between the work environment and the caregiver’s ethical sensitivity. Studies reveal a need to develop evidence-based support both on the organizational and individual levels to support caregivers’ ethical competence.^29,36^ The ethical climate of a care organization shapes the care given and the various aspects that promote an ethical environment and care culture in which open and honest communication is promoted.^30,37,35,29,38^ Accordingly, unless the value of compassion in care work is recognized on the organizational level the work culture at an organization can have a negative impact on the organization’s ethical climate, and a high workload in relation to understaffing can also have a negative impact on the existence of compassion in care work.^38,36^

The literature review above shows that most studies have primarily focused on caregivers’ or clients’ experiences of ethical sensitivity and compassion. To address the research gap, that is, care leaders’ perspectives on the subject matter, an understanding of home care leaders’ perceptions of ethical sensitivity and compassion in a home care context was sought.

## Aims

The aim of the study was to explore home care leaders’ perceptions of ethical sensitivity and compassion associated with care quality in home care.

### Theoretical perspective

Eriksson’s caritative caring theory was used as a theoretical perspective, where the human being is at the center of caring,^19^ and a compassionate view of humanity is seen as the basis for a caritative caring.^39^ Ethics is a fundamental part of caritative caring theory, which means that the caregiver sees reality from the patient’s perspective and treats the patient with dignity so that the patient feels confirmed as the unique human being he/she is.^40^ Caritas, love, and mercy are considered the basic motive for all caring and the person who provides the caring, that is, the caregiver, holds the love of the other as a fundamental value.^19^ Compassion is included as a motive for caring when the caregiver strives to alleviate human suffering^40^ and compassion becomes visible in acts, seen as the caregiver’s willingness to help through a selfless act of love for a suffering person in order to reduce suffering.^40,19^ It is the suffering human being that motivates caring on a deeper level and it is in the true compassion where love and suffering meet that real care arises, where with the power of love one can care and alleviate suffering.^19^ According to Eriksson,^40,19^ true caring in compassion presupposes the courage to take responsibility for and sacrifice a part of oneself for the sake of the other. Compassion is born of seeing the suffering of others^41^ and can be viewed as sensitivity to another human being’s suffering that provides the impetus to fight and try to alleviate the suffering.^19^ Compassionate caregivers who with authenticity allow themselves to be touched by others’ suffering are considered good caregivers who make patients feel worthy.^42^ Deeper human knowledge helps the caregiver see each human being as unique, and Arman^39^ believes that a caregiver can practice awareness where each patient is seen as a fellow human being with whom the caregiver has a relationship, which also allows the caregiver to recognize the vulnerability that belongs to life. In such a care communion, where the patient and caregiver meet in reciprocity, natural humanity and professional care have been brought together so that the caring meeting between caregiver and patient gives meaningfulness to both.^43^

## Methodological aspects

A qualitative explorative design was used. The data material consisted of texts from in-depth interviews with ten home care leaders. The method used was qualitative content analysis.^44^

### Data material, collection, and analysis

Semi-structured interviews were conducted with 10 home care leaders. Participants were recruited electronically via an invitation sent by e-mail to several municipalities and a welfare area, in which those interested in participating in the study were asked to personally contact the researchers. The researchers’ contact information was included in the invitation. All participants were women and their work experience as leaders in home care varied between 2 and 13 years. Most participants also had experience from other areas of social welfare and healthcare. Given the ongoing COVID-19 pandemic, nine interviews were held via videography and one interview via telephony. The interviews lasted for about 30–60 min. All interviews were taped and transcribed verbatim.

The collected interview data were then analyzed with qualitative content analysis.^44^ This meant that the researchers paid attention to the nuances and underlying meanings of the textual content of the data. The data material was read several times, after which the content was analyzed into units of meaning. These units of meaning were then condensed, encoded, and grouped, with the aim to find themes that explained the content of the data material, *expressed as an overall theme and subthemes.*

### Ethical considerations

The study was conducted in accordance with good scientific practice by following the ethical guidelines of the Declaration of Helsinki^45^ and the Finnish Advisory Board of Research Ethics^46^ during all stages of the study. Research ethics permission was applied for from the Research Ethics Board at the university setting where the researchers were employed. Research permission to conduct the interviews for the study was granted by the four included organizations from which the participants were recruited. Written informed consent regarding participation in the study and handling of data for research purposes was obtained from all participants. The participants received information about the study both orally and in writing before the interviews began.

## Findings

**Figure 1.**
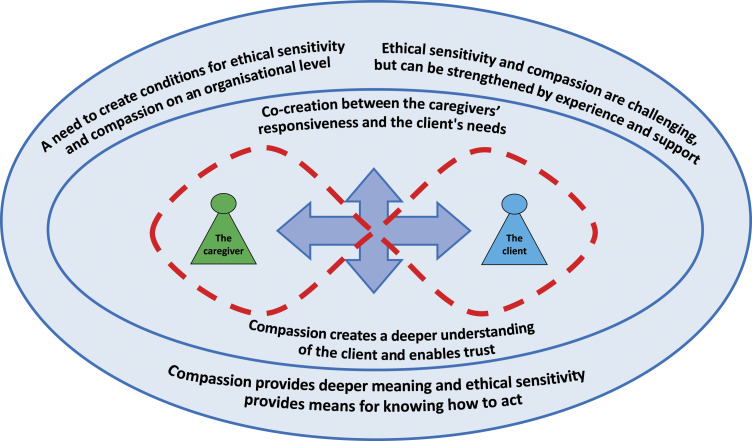
Overview of the findings.

One overall theme and four subthemes were found. The overall theme was: Compassion provides deeper meaning and ethical sensitivity provides means for knowing how to act. The four main themes were: Co-creation between the caregivers’ responsiveness and the client’s needs, Compassion creates a deeper understanding of the client and enables trust, Ethical sensitivity and compassion are challenging, but can be strengthened through experience and support, and A need to create conditions for ethical sensitivity and compassion on the organizational level. For an overview of the findings, see Figure 1.

## Compassion provides deeper meaning and ethical sensitivity provides means for knowing how to act

This overall theme showed that when ethical sensitivity and compassion are present in care, the home care leaders relate that a trusting relationship between the client and the caregiver can be created. The leaders highlight the caregivers’ approach to treatment as well as trust as co-creators of a trusting relationship where ethical sensitivity and compassion are present, and they see these as important factors that can raise the quality of care. The organization and the leaders create the conditions for a permissive environment where ethical sensitivity and compassion can be seen as resources. According to the home care leaders, aspects that are seen to be of particular importance on the part of the leaders and the organization include that the caregivers receive sufficient support by being seen, having their needs recognized and are given space for discussion in order to manage their emotions and being allowed to feel them. Leadership is also described as being put to the test in terms of the leader’s ability and ethical sensitivity to be able to distinguish between the employees who manage independently and those who need support. In this way, according to one of the home care leaders, they can also be seen as role models by having ethical sensitivity and compassion present in their approach in the workplace. Leadership in home care means taking into account employees as a group as well as the needs of the individual caregiver. Since caregivers in home care work individually at the client’s home, the home care leaders believe that it is of particular importance to have a strong and good collaboration within the team for the care to be qualitative. The home care leaders highlight this and mention that they have close-knit teams in which they help and support each other.

Ethical sensitivity and compassion constitute competencies that are tied to one’s personality and are something most people who seek to work in the field of care have. However, these competences can even be developed and strengthened through experience of nursing care and/or support from colleagues or a care organization. Ultimately, however, the responsibility for ensuring that ethical sensitivity and compassion are actually present in the care given in a client’s home lies with the caregiver. It is the caregiver who, by being responsive to the client and his/her needs, can see the client’s suffering and demonstrate compassion. Ethical sensitivity enables the caregiver to relate ethically to each unique situation as though it were “new”, and by being sensitive to the client’s stated or unexpressed care needs and desires, the caregiver can form an idea of how to show compassion through actions and attitude.

*You have to read these situations individually… if I come to your house and I see that you are suffering, then one time maybe it’s okay for me to take you by the hand but the other time it may not be okay that you are so close but you listen more then and try to increase your understanding*. (P5).

## Co-creation between the caregivers’ responsiveness and the client’s needs

This subtheme concerns compassion as interaction (co-creation) between the caregiver’s responsiveness and the client’s needs and desires, both stated and unspoken. This also included treating the patient with dignity and showing respect for the client and client’s home, for example, acting humbly, paying attention to the client’s wishes, and respecting the client’s home, rules, routines, and habits.

Ethical sensitivity and compassion are components that home care leaders consider to be essential in home care. Home care entails entering another person’s “domain”, thus ethical sensitivity must constitute a cornerstone of such care; care can suffer if the caregiver does not demonstrate ethical sensitivity.

*Ethical sensitivity applies to everything that they do with the client, it’s in every moment I think* (P8)

*Ethical sensitivity is quite crucial to whether or not there will be continued care—if clients feel that we are violating their life situation or their lives in their own home in some way, they can end home care even though the need exists*. (P9)

The caregiver’s ethical sensitivity implies responsiveness to the needs of the client and can improve the caring encounter: *As a caregiver, you need to find this boundary of what I can or cannot do, that you have to listen to the client, read from the client what is working right now.* (P4). Ethical sensitivity is described as being open in the caring encounter, being able to discern client nuances, paying attention to and encountering the client’s needs with compassion, and showing respect for the client and his/her right to self-determination. This means responsiveness to both the clinical needs needed for good care and also the more personal needs that can be significant on a more emotional level, for example, a smile or a touch. Ethical sensitivity occurs as a prerequisite for interaction between caregiver and client, where the caregiver is responsive to the client’s needs and can discern what the client perceives as meaningful. Ethical sensitivity is preceded by compassion for the human being and compassion is seen as the basis that enables interaction. Through ethical sensitivity, clients can feel that they are being listened to, heard, and allowed to decide for themselves about their care, their home, and their life, which is important for the quality of care. Care needs to be based on the client’s specific needs with the client at the center of care; the caregiver should have a person-centered approach, which is made possible by the caregiver’s ethical sensitivity.

Ethical sensitivity is described as being ever-present and is especially important when it comes to care in the client’s home. Ethical sensitivity is explained as entering the client’s home in a respectful manner, maintaining one’s professional role, treating the client well, and respecting the client’s integrity and right to self-determination. Good treatment, respect for clients, their integrity, and home and self-determination are highlighted as important. That ethical sensitivity is present when it comes to these aspects is seen as crucial for the continuation of care. Being able to show respect for the client through using ethical sensitivity is also described as encountering clients on their level and respecting their values, even if those differ from the caregiver’s own: *You have to remember that all clients have … the right of self-determination, that it is their life and as a caregiver you can’t enter their home without ethical sensitivity.* (P4)

## Compassion creates a deeper understanding of the client and enables trust

This subtheme describes compassion as seeing and confirming the client’s suffering. According to the home care leaders’ views, compassion is clearly interrelated with ethical sensitivity in terms of understanding the clients’ situation and conveying compassion by listening, being present, showing care, touch, and love—actions that can alleviate suffering. According to the home care leaders, compassion conveyed with ethical sensitivity creates trust and warmth and is consensus-based care.

The caregiver values the client as a fellow human being by showing compassion for the client through bearing, action, and being fully present in the moment: *That the client leaves with the feeling that they have been heard, seen and feel that “I am important to someone, they care about me and want the best for me.”* (P7) This caring relationship is important because home care visits may be the only human contact that some clients have during a day. The caring relationship and the co-creation in care that the relationship entails make the care important for both the caregiver and the client, which also increases the value of the care, according to the home care leaders.

Clients experience more suffering if compassion is not present. If the client suffers and no one takes the time to show compassion, it creates an even greater suffering for the client, according to the home care leaders, that directly affects care quality negatively because clients may experience worry or insecurity so that the client’s care needs increase. Home care leaders consider compassion to be central to good care quality: *Compassion is essential* (P10). Compassion can be expressed through a smile, a touch or a word while others explain it as actively listening, being there, or giving of their time. Home care leaders stress that care with compassion can give the client security and thus also affect the ability to continue living at home. Compassion is about facing the client’s suffering, trying to interpret what the client wants and needs, and taking measures that can alleviate the client’s suffering, for example, showing compassion at the level the client needs at that particular moment: *Sometimes it can be about just sitting together for a while in silence* (P9).

That decisions are made with the client’s needs and desires in focus provides greater opportunities for the client to feel involved. This is explained as the caring encounter being made a shared experience for the caregiver and their clients, where the caregiver actively listens, sees, and pays attention to the clients, takes them through different care situations, and the clients dare to rely on their own problem-solving skills and on the caregiver’s professionalism: *… so that it becomes an important experience—that we [the patient and the caregiver] have done this together* (P2). By including ethical sensitivity and compassion in these situations, trust can be created in the caring encounter, and this enables a trusting care relationship. According to the home care leaders, care runs more smoothly when it is done in agreement between caregivers and clients. Ethical sensitivity and compassion allow the caregiver to better meet the client’s needs in the caring encounter, which as indicated by the home care leaders enables care quality: *If the caregiver and the client have a good relationship, the client may dare to say everything and not hold back if there is something wrong.* (P8) Care that is given with compassion can also result in a decreased need for home care: *They have had this ability to provide care so that the client felt safe in their new role with their illness and can now cope with less home care* (P2).

## Ethical sensitivity and compassion are challenging, but can be strengthened by experience and support

This subtheme refers to ethical sensitivity and compassion as competencies that can be developed and the need for support. Home care leaders view compassion as feeling with someone in their suffering and vulnerability. Most people who have chosen the nursing profession have a certain level of innate ethical sensitivity and compassion, but still need to develop skills and need support to express compassion. Through life experience, professional experience and support in various forms, caregivers can develop their ethical sensitivity and compassion.

As indicated by the home care leaders, challenges are partly that there are caregivers who often want more than the resources allow, which can lead to distress and personal suffering in the caregiver, and partly that some caregivers do not have sufficient expertise in this area because of inexperience, personality, lack of commitment or unwillingness to learn. However, in reference to the caregivers’ ethical sensitivity and compassion as competencies, most of the home care leaders describe their personnel in positive terms.

Ethical sensitivity and compassion are seen by the home care leaders as abilities linked to one’s personality that can be developed and strengthened through knowledge and work experience. In order to have ethical sensitivity, several of the home care leaders believe that the caregivers first need to feel secure in their professional role. Through experience, home care leaders see that caregivers also learn to deal with feelings of compassion and see these as something that belongs to a caregiver: *I think that everyone who applies to the care sector has ethical sensitivity and compassion* (P4). The home care leaders agree that the level of competence regarding ethical sensitivity and compassion varies between different caregivers, which also means that the care provided is not equal. This is described as having an impact on care as follows: *The quality of care is probably affected if the caregiver does not have ethical sensitivity or compassion because then the caregiver does not see if the client is unwell or suffering* (P6).

Ethical sensitivity is seen as a skill that develops primarily with job experience while compassion is more linked to the personality. Some leaders see compassion as an innate trait formed when growing up so that some have the ability to feel compassion while others find it more difficult to detect a client’s suffering.

For the home care leaders, how the caregiver deals with feelings of compassion crucially impacts the quality of care. One of the home care leaders expresses it as follows: *If you are a caregiver, you give of yourself to another person, then you also need to have the tools to know how to cope with what you receive in return* (P2). The challenges in home care can quickly become mentally burdensome when it comes to balancing the professional role and dealing with feelings of compassion. This is because it may not only be the client’s illness and ill health that may have given rise to the need for home care, but the caregiver’s visit to the client’s home also sometimes includes difficult home conditions, loneliness and financial difficulties: *There are life stories that touch you far too much where you can’t influence to the extent you would like, which means that the work eats you up from within* (P1). Healthcare professionals need to more openly discuss difficult work encounters and the emotions that may arise from such, for example, with their colleagues or a professional coach or psychologist, because they need support both in dealing with and in conveying ethical sensitivity and compassion. It is important that the colleagues discuss difficulties so that the caregivers can better manage their own compassion. More discussion and support around ethical sensitivity and compassion would be needed but it is difficult to put in more support measures with the current scarce resources in home care.

Ethical sensitivity and compassion are important factors in home care because if these are lacking the result may be an increased need for care: *If the client feels that he does not receive any compassion, it can lead to a decrease in mood, the feeling drops and then the need for care can increase—there will be a deterioration in both directions so that the client feels worse and the caregiver’s job increases, it affects everything* (P4).

## A need to create conditions for ethical sensitivity and compassion on the organizational level

This subtheme is about the roles of the organization and home care leaders in directly impacting the ability to see ethical sensitivity and compassion as resources in home care by maintaining a compassionate work environment, ensuring the well-being of caregivers and meeting clients’ needs with adequate resources. According to the home care leaders, a lack of resources can affect caregivers’ ability to give time, consideration, and compassion to clients, which can cause caregivers distress. That sufficient and proper support is provided for caregivers is therefore seen by the home care leaders as very important and as simultaneously enhancing caregivers’ well-being. How well these matters are supported affects the care provided in terms of quality. The caregivers usually have the will to nurture with ethical sensitivity and compassion but the resources provided by the organization are not always sufficient.

Together with its leader, an organization can influence by creating a supportive environment that encourages ethical sensitivity and compassion. The presence of ethics and compassion in home care is influenced by the work environment and the work community and is visible in the interaction in the team. This is described as listening to each other, giving one another advice, and having a good social interaction where everyone is taken into account, which also supports team collaboration. The home care leaders themselves have an important role in creating a work environment where values, for example, ethical sensitivity and compassion, are included and highlighted in the work climate*.*

Generating compassion during the home care visit can, at least for the moment, provide warmth and reduce a client’s sense of loneliness. Lack of resources in home care can unfortunately affect the caregiver’s ability to give time, consideration, and compassion to the client: *Having the professional role while having compassion, being flexible and trying to help as much as possible and with the lack of resources, this is difficult to balance and then it can quickly become mentally burdensome* (P4). If the planned time for the visit is insufficient, it may entail that the caregiver is responsive to the client’s needs but cannot meet them. This can then, according to the home care leaders, create distress and even possibly burnout over the long term in caregivers because the caregivers feel compassion but cannot take the time to convey this compassion to the client. Home care leaders also see risks with resource scarcity in that care measures can become routine and the client’s needs no longer are placed at the center, which negatively affects the care quality. According to the home care leaders, over the past decade home care has changed from being genuine care with time for the client to becoming “piecework”. Home care leaders stress that the lack of resources and urgency in home care can make caregivers less responsive to clients’ needs, which can lead to problems or mistakes and lead over the long run to increased care needs. While home care leaders wish for more resources in home care so that compassion can remain a part of the care being given, the reality is that resources are decreasing while the number of clients who require home care services is increasing (because it is expected that care should be given in the home and not an institutional setting).

Caregivers’ well-being is important and linked to their ability to demonstrate ethical sensitivity and feel compassion for clients. Ethical sensitivity and compassion are not only vital for clients and care but also add value to caregivers in their professional role. To feel and show compassion involves interaction with the client and ethical sensitivity requires the caregiver to be truly responsive and actively listen to the client, which creates a relationship that can give both the caregiver and client a sense of meaningfulness. Therefore, the home care leaders also see it as important that not only the organization but even leaders themselves support caregivers. To cope with this, time is also required for recovery. Little things bring meaningfulness to home care: *One little thing can make such a big difference in someone’s well-being, by being available and showing that you care* (P9). The role of the organization is to support caregivers’ well-being by meeting their needs and providing adequate resources in home care. Discussion of ethical sensitivity and compassion in the workplace can make their presence more evident in daily work. Workplace training on the subject, workshops or regular ethical discussions (ethical rounds) are also things that home care leaders highlight as supportive measures that might have a positive impact on caregivers’ competence regarding ethical sensitivity and compassion: *Having ethical discussions… I’m a great believer in discussing in the team, in sharing what you’ve been through… you grow as a person both privately and in your professional role* (P2).

## Discussion

The aim of the study was to explore home care leaders’ perceptions of ethical sensitivity and compassion associated with care quality in home care. The study showed that home care leaders viewed ethical sensitivity and compassion as a competence that caregivers in home care should have because such competencies create deeper meaning in the care relationship for both the caregiver and client and also increases the value of care. If caregivers are not responsive to clients’ needs, high-quality care cannot be achieved.^47^ This is consistent with previous research that has shown that caregivers’ ethical sensitivity^47,30,35^ and compassion in care^9,18,15^ can contribute to increased value of care and care quality.^47^ The presence of compassion improves care outcomes for clients^48^ and client safety^49^ and even has positive effects on caregivers, who through a sense of appreciation can increase their motivation to provide good care.^48,18^ Compassion is also linked to lower levels of burnout in caregivers and a higher sense of satisfaction in the workplace.^50^ Compassion in the caring encounter consciously draws attention to the needs of the client^15^ and facilitates interaction between the caregiver and client that can improve care.^51^ Ethical sensitivity and responsiveness preserve patient dignity.^34^

In this study, ethical sensitivity was understood to be interrelated with compassion; through ethical sensitivity the caregiver can be responsive to the client’s needs and convey compassion in actions that can alleviate suffering. This is in line with Tehranineshat et al.,^14^ who describe compassionate care as professional care that occurs through clinical expertise, ethical values, and sensitivity to needs. Hemberg and Bergdahl^34^ also underline that ethical sensitivity and responsiveness in co-creative encounters with the client can help caregivers balance their actions in the moment and base the design of care measures on the client’s needs. Sinclair et al.^49^ suggest that the verbal and nonverbal communication that takes place between the caregiver and client includes an element of compassion, where the caregiver and client together create a caring relationship by acknowledging and creating an understanding of the client’s needs. Durkin et al.^56^ highlight that compassion includes motivation that comes from the caregiver’s inner will to alleviate the client’s suffering, a relationship that includes the use of emotionally engaging communication to create an understanding of the client’s suffering, an active presence where the caregiver is responsive to the client’s needs, and action to alleviate the client’s suffering that is based on the client’s individual needs. Likewise, as seen in this study, ethical sensitivity and compassion are closely intertwined in this regard. According to the home care leaders included in this study, ethical sensitivity is the means whereby the caregiver responds to the client in order to discover what *is* significant for the client, while compassion is what *makes* the care significant for both the caregiver and client. These findings are in accordance with Eriksson’s^19^ caritative caring theory; in the caring communion, genuine dialogue and the consideration of emotions occurs between caregiver and client, which creates meaning for both.

This study showed that ethical sensitivity and compassion are challenging but are abilities that can be developed and strengthened through knowledge and work experience. However, earlier epistemological research is not conclusive on the matter. Saunders^10^ views compassion as a quality that can be developed in all people. Bond et al.^52^ also see compassion as one of the natural qualities of the human being and argue that compassion consequently cannot be taught but can instead be developed through repeated behavior and observation. Compassion is also described as an individual ability based on personal beliefs, values, knowledge, and attitude toward others.^53^ Ethical sensitivity can be improved by developing one’s empathic ability^54^ or through knowledge and education.^30^ Weaver et al.^55^ suggest that ethical sensitivity can be developed in professional practice in situations that enable the caregiver to recognize and respond to the client’s suffering and vulnerability. As seen in this study, caregivers can develop certain attributes of ethical sensitivity, for example, the ability to be responsive, receptive, and motivated to alleviate suffering; interpret perspectives; and, through commitment, decide on the appropriate course of action. Also seen in findings of this study was that ethical sensitivity and compassion primarily develop through professional experience, support from care leaders in the form of discussions, and through open and honest discussion between colleagues. Kim and Lee^37^ also found that care leaders should implement programs to support compassion in care. Van der Cingel^15^ advocates the formal placement of compassion as a leading concept in healthcare practice, where compassion is highlighted as an empowering characteristic. Through a focus on relationships and motivated leadership in the form of supporting and strengthening a commitment to conversations and dialogues that contain more substance and reflection, compassion in care work can be developed and have a positive impact on care quality.^23^

As indicated by the home care leaders in this study and seen in the findings, caregivers in home care demonstrate ethical sensitivity and compassion but risk feelings of distress without sufficient support. Lee and Seomun^50^ suggest that compassion competence is a predictive factor for professional quality of life and should be improved to reduce the risk of burnout among caregivers. Gustafsson and Hemberg^57^ underscore that caregivers can experience compassion fatigue because of high exposure to client suffering that requires great empathic energy and compassion in combination with various (negative) work organization and/or work community aspects. Ghafourifard et al.^53^ emphasize that administrative managers in healthcare should develop and adopt strategies to develop programs that can improve compassionate skills. In a compassionate organization, caregivers can support each other, which simultaneously improves compassion competence and thereby improved care quality, client experience, and client safety as well as reduces caregivers’ stress and risk of burnout.^37^ In this study, the home care leaders felt that it can be challenging for caregivers to discern which limits should be placed on compassion so that their compassion does not become personal suffering or affect their ability to provide care. As seen in the findings, both caregivers’ ability to manage emotions and whether their needs are recognized and opportunities for discussion given impact how they manage and convey ethical sensitivity and compassion. Caregivers who cannot manage compassion may distance themselves from clients who need emotional support^58,59^ and difficulties managing compassion may influence caregivers’ attitudes when performing care measures.^58,57^ Research shows that there are even challenges associated with being close to suffering and/or caregivers’ fear of being touched as well as a fear of own reactions and powerlessness over such.^21^ Wiklund Gustin^21^ describes how caregivers need to feel satisfaction and self-compassion to face such fears, which entails acknowledging these feelings. Other challenges that emerged in this study’s findings were that because caregivers’ competencies in ethical sensitivity and compassion varied, the care being provided was not equal. Sufficient time, job satisfaction, a sense of support, and appreciation affect caregivers’ ability to deliver compassionate care.^60^ The study also revealed that caregivers need support from leaders and adequate and sufficient resources, for example, in the form of time, to realize ethical sensitivity and compassion in care encounters. However, it was also revealed in the findings that the resources for home care are decreasing at the same time that the need for home care is increasing. The resources needed are not about the time needed to (routinely) perform actions per se but are instead more about the time caregivers need to demonstrative responsiveness toward clients’ suffering and emotionally engage.^56^ Research shows that opportunities for support and training are often reduced when workload is high, which negatively affects caregivers’ ability to provide quality and compassionate care.^60^ Nevertheless, despite such challenges, care leaders are responsible for supporting caregivers in difficult situations, for example, when suffering occurs, so as to enable caregivers to “pass on” care to clients.^61^ Leaders should serve as role models in the sense that they should be responsive toward caregivers and have the competence to convey compassion through a sympathetic and understanding approach.^61^ Likewise, as seen in this study, leaders should be sensitive to staff’s well-being because suffering was seen to affect caregivers’ ability to show clients compassion.

Compassion in care is influenced by both individual^53^ and organizational factors.^60,61^ A heavy workload is seen as an obstacle to compassion in healthcare^53^ and a lack of ethical knowledge and professional experience are seen as barriers to ethical sensitivity.^30^ The lack of resources described in this study potentially inhibits caregivers from providing good care that includes ethical sensitivity and compassion. Each organization, including its leaders, should provide sufficient support so as to facilitate ethical sensitivity and compassion and should consider such to be a resource that raises care quality. Salmela et al.^63^ highlight that the basis of care is formed from the care culture present, seen through its traditions and habits. Lown^48^ proposes the integration of compassion into organizations in such a manner that it becomes a natural feature of everyday work while Huang et al.^30^ considers the application of ethical knowledge in practice to be a factor that could develop ethical sensitivity. Christiansen et al.^64^ maintain that a leadership that supports and ensures caregivers’ well-being simultaneously contributes to more compassionate care. Supporting caregivers to be compassionate in practice also entails an organization and its leaders acknowledging that caregivers need time to convey compassion,^56^ which is in line with the needs identified in this study. Compassion in caregivers even means that they have a deep understanding of their colleagues, which creates the opportunity to not only receive but also provide additional support to those struggling with responsibility issues around clinical practice while even improving community in the workplace.^37^ Factors that can facilitate such are effective leadership, collegial support, a healthy work environment,^53^ skill development through education,^62^ communication, direct support for personal development, job satisfaction, as well as adequate time for each client, realized through a sufficient number of caregivers and time for the execution of care itself.^60^ This study shows that an organization and its leaders play a significant role in creating a work culture where ethical sensitivity and compassion are present. If an organization integrates a focus on compassion and the various aspects of compassion into time management in care, care work becomes more attractive^52^ and care outcomes can improve.^37,36^ Furthermore, compassion can increase care quality and help clients feel more secure, and caregivers may also find increased meaningfulness in their work.^53^ Moreover, through support, staff can experience enhanced well-being and commitment.^60^

### Strengths and limitations

The participants were chosen based on their experiences as home care leaders, through which they engaged in collaboration with staff and experienced close contact with clients and clients’ relatives.

The inclusion of home care leaders as research participants, therefore, can be said to yield a comprehensive perspective on the research subject investigated, experiences of ethical sensitivity, and compassion in home care. Most study participants had lengthy experience of the nursing profession and even previous professional experience as a nurse, which can be considered a strength. Another strength is that the participants had varied professional backgrounds and different educational degrees. Participants were sought from different areas of Finland. However, due to the COVID-19 pandemic and a resultant lack of healthcare resources during the data collection period, the sample size was small. Nevertheless, the data were seen to be rich with detailed information on the research topic. The study can therefore be considered of importance on the individual, societal, and professional levels and can be considered to be of use to those working in home care as well as those who train caregivers.

## Conclusions

An understanding of compassion and ethical sensitivity as a source of strength and as linked to care quality in a home care context was derived. A focus on how to increase compassion and ethical sensitivity in home care settings is recommended in future research, including how healthcare professionals’ compassion and ethical sensitivity can be adequately supported.

## References

[1] Kristinsdottir IV Jonsson PV Hjaltadottir I, et al. Changes in home care clients’ characteristics and home care in five European countries from 2001 to 2014: comparison based on InterRAI - home care data. BMC Health Serv Res 2021; 21(1): 1–1177. DOI: 10.1186/s12913-021-07197-3. 34715850 PMC8555210

[2] ETENE, Riksomfattande etiska delegationen inom social- och hälsovården. Den etiska grunden för social- och hälsovården (ETENE-publikationer 33). Social- och hälsovårdsministeriet. https://etene.fi/documents/1429646/1571620/Publikation+33+Den+etiska+grunden+f/C3/B6r+social-+och+h/C3/A4lsov/C3/A5rden,+2011.pdf/3cd3621e-5301-43d7-9eeb-5f6aecf84f5e/Publikation+33+Den+etiska+grunden+f/C3/B6r+social-+och+h/C3/A4lsov/C3/A5rden,+2011.pdf?t=1439806054000 (2011).

[3] American Nurses Association (ANA). Guide to the code of ethics for nurses with interpretive statements: development, interpretation, and application. 2nd ed. 2015.

[4] ETENE, Riksomfattande etiska delegationen inom social- och hälsovården. Care ethics. In the autumn of life. (In Swedish: Vårdetik. På ålderns höst). ETENE-publikationer 21. Social- och hälsovårdsministeriet. https://etene.fi/documents/1429646/1571620/Publikation+22+ETENEs+rapport+om+v/C3/A5rdetik+p/C3/A5+/C3/A5lderns+h/C3/B6st/2C+2008.pdf/bf7c560e-0569-48b1-8eec-e42c098c15e4/Publikation+22+ETENEs+rapport+om+v/C3/A5rdetik+p/C3/A5+/C3/A5lderns+h/C3/B6st/2C+2008.pdf (2008).

[5] Silén M. Quality development in nursing. Nurses’ professional responsibilities. (In Swedish: Kvalitetsutveckling inom omvårdnad. Sjuksköterskans professionella ansvar). Etiska aspekter på omvårdnad och prioriteringar. In:Hommel A Andersson Å (Red) Lund: Studentlitteratur, 2018.

[6] Hemberg J Nyqvist F Näsman M Meaningfulness in Daily Life among Frail Older Adults in Community Living in Finland. Health Promotion International 2022; 37(2). DOI: 10.1093/heapro/daab087. PMC9053457 34339504

[7] Hemberg J Syrén J Hemberg H Ethical Leadership in a New Light – as Described by Leaders in Public Health Care. International Journal for Human Caring 2018; 22(4): 179–188. DOI: 10.20467/1091-5710.22.4.179.

[8] Aitamaa E Leino-Kilpi H Iltanen S, et al. Ethical problems in nursing management: the views of nurse managers. Nurs Ethics 2016; 23(6): 646–658. DOI: 10.1177/0969733015579309. 25899724

[9] Fotaki M. Why and how is compassion necessary to provide good quality healthcare? Int J Health Policy Manag 2015; 4(4): 199–201. DOI: 10.15171/ijhpm.2015.66. 25844380 PMC4380560

[10] Saunders J. Compassion. Clin Med (London, England) 2015; 15(2): 121–124. DOI: 10.7861/clinmedicine.15-2-121. PMC4953728 25824061

[11] Eriksson K. The suffering human being. (In: Swedish: Den lidande människan). Liber, 1994.

[12] Levinas E. Useless suffering. In: Bernasconi R Wood D (eds) The provocation of levinas: rethinking the other. London: Routledge, 1988.

[13] International Council of Nurses (ICN). Code of Ethics for Nurses. https://www.icn.ch/sites/default/files/inline-files/2012_ICN_Codeofethicsfornurses_/20eng.pdf (2012).

[14] Tehranineshat B Rakhshan M Torabizadeh C, et al. Nurses’, patients’, and family caregivers’ perceptions of compassionate nursing care. Nurs Ethics 2019; 26(6): 1707–1720. DOI: 10.1177/0969733018777884. 29898620

[15] van der Cingel M. Compassion: the missing link in quality of care. Nurse Educ Today 2014; 34(9): 1253–1257. DOI: 10.1016/j.nedt.2014.04.003. 24856582

[16] Shea S. Is it possible to develop a compassionate organization? Comment on “why and how is compassion necessary to provide good quality healthcare?”. Int J Health Policy Manag 2015; 4(11): 769–770. DOI: 10.15171/ijhpm.2015.119. 26673338 PMC4629703

[17] Strauss C Lever Taylor B Gu J, et al. What is compassion and how can we measure it? A review of definitions and measures. Clin Psychol Rev 2016; 47: 15–27. DOI: 10.1016/j.cpr.2016.05.004. 27267346

[18] Sinclair S Beamer K Hack TF, et al. Sympathy, empathy, and compassion: a grounded theory study of palliative care patients’ understandings, experiences, and preferences. Palliat Med 2017; 31(5): 437–447. DOI: 10.1177/0269216316663499. 27535319 PMC5405806

[19] Eriksson K. Caring science. The science of caring. About the timeless in time. (In: Swedish: Vårdvetenskap. Vetenskapen om vårdandet. Om det tidlösa i tiden). Liber, 2018.

[20] Hemberg J Wiklund Gustin L Caring from the heart as belonging – nurses experiences of mediating compassion. Nurs Open 2020; 7(2): 660–668. DOI: 10.1002/nop2.438. 32089865 PMC7024612

[21] Wiklund Gustin L. Humanity and ”compassion”. (In Swedish: Medlidande och ”compassion”). In: Wiklund Gustin L Bergbom I (eds) Caring science concepts in theory and practice (In Swedish: Vårdvetenskapliga begrepp i teori och praktik). 2nd ed. Lund: Studentlitteratur, 2017, pp. 353–363.

[22] Bramley L Matiti M. How does it really feel to be in my shoes? Patients’ experiences of compassion within nursing care and their perceptions of developing compassionate nurses. J Clin Nurs 2014; 23(19–20): 2790–2799. DOI: 10.1111/jocn.12537. 24479676 PMC4263156

[23] Dewar B Cook F. Developing compassion through a relationship centred appreciative leadership programme. Nurse Educ Today 2014; 34(9): 1258–1264. DOI: 10.1016/j.nedt.2013.12.012. 24461906

[24] Kagan SH. Compassion. Geriatr Nurs (New York) 2014; 35(1): 69–70. DOI: 10.1016/j.gerinurse.2013.11.006. 24365227

[25] Wiklund Gustin L Wagner L. The butterfly effect of caring - clinical nursing teachers’ understanding of self-compassion as a source to compassionate care. Scand J Caring Sci 2013; 27(1): 175–183. DOI: 10.1111/j.1471-6712.2012.01033. 22734628

[26] Bergbom I Nåden D Nyström L. Katie Eriksson’s caring theories. Part 1. The caritative caring theory, the multidimensional health theory and the theory of human suffering. Scand J Caring Sci 2021; 36. DOI: 10.1111/scs.13036. 34609017

[27] Hemberg J The dark corner of the heart – understanding and embracing different faces of suffering as portrayed by adults. Scandinavian Journal of Caring Sciences 2017; 31(4): 995–1002. DOI: 10.1111/scs.12424. 28239889

[28] Weaver K. Ethical sensitivity: state of knowledge and needs for further research. Nurs Ethics 2007; 14(2): 141–155. DOI: 10.1177/0969733007073694. 17425144

[29] Poikkeus T Numminen O Suhonen R, et al. A mixed-method systematic review: support for ethical competence of nurses. J Adv Nurs 2014; 70(2): 256–271. DOI: 10.1111/jan.12213. 23865484

[30] Huang FF Yang Q Zhang J, et al. Chinese nurses’ perceived barriers and facilitators of ethical sensitivity. Nurs Ethics 2016; 23(5): 507–522. DOI: 10.1177/0969733015574925. 25825415

[31] Milliken A Grace P. Nurse ethical awareness: understanding the nature of everyday practice. Nurs Ethics 2017; 24(5): 517–524. DOI: 10.1177/0969733015615172. 26659025

[32] Lechasseur K Caux C Dollé S, et al. Ethical competence: an integrative review. Nurs Ethics 2018; 25(6): 694–706. DOI: 10.1177/0969733016667773. 27694548

[33] Borhani F Abbaszadeh A Mohsenpour M. Nursing students’ understanding of factors influencing ethical sensitivity: a qualitative study. Iranian J Nurs Midwifery Res 2013; 18(4): 310–315. PMC3872867 24403928

[34] Hemberg J Bergdahl E Ethical Sensitivity and Perceptiveness in Palliative Home Care through Co-creation. Nursing Ethics 2020; 27(2): 446–460. DOI: 10.77/0969733019849464. 31280654

[35] Kulju K Stolt M Suhonen R, et al. Ethical competence: a concept analysis. Nurs Ethics 2016; 23(4): 401–412, DOI: 10.1177/0969733014567025. 25670176

[36] Straughair C. Exploring compassion: Implications for contemporary nursing. Part 2. Br J Nurs (Mark Allen Publishing) 2012; 21(4): 239–244. DOI: 10.12968/bjon.2012.21.4.239. 22398938

[37] Kim C Lee Y. Effects of compassion competence on missed nursing care, professional quality of life and quality of life among Korean nurses. J Nurs Manag 2020; 28(8): 2118–2127. DOI: 10.1111/jonm.13004. 32166797

[38] Valizadeh L Zamanzadeh V Dewar B, et al. Nurse’s perceptions of organisational barriers to delivering compassionate care: a qualitative study. Nurs Ethics 2018; 25(5): 580–590. DOI: 10.1177/0969733016660881. 27514741

[39] Arman M. The world of the patient - when the human being becomes a patient. Seeing the patient as a fellow human being. (In Swedish: Patientens värld - när människan blir patient. Att se patienten som en medmänniska). In: Arman M Dahlberg K Ekebergh M (eds) Theoretical foundations of caring (In Swedish: *Teoretiska grunder för vårdande*). Liber, 2015, pp. 76–81.

[40] Eriksson K. Towards a caritative caring ethics. (In Swedish: *Mot en caritativ vårdetik*). Åbo Akademi, 1995, pp. 9–35.

[41] Arman M. Suffering. (In Swedish: Lidande). In: Wiklund Gustin L Bergbom I (eds) Caring science concepts in theory and practice. (In Swedish: *Vårdvetenskapliga begrepp i teori och praktik*). 2nd ed. Lund: Studentlitteratur, 2017, pp. 213–222.

[42] Söderlund M. Caring. (In Swedish: Vårdande. In: Wiklund Gustin L. Bergbom I (eds). Caring science concepts in theory and practice. (In Swedish: *Vårdvetenskapliga begrepp i teori och praktik*). 2nd ed. Lund: Studentlitteratur, 2017, pp. 295–304.

[43] Arman M. Compassion and empathy – the reflection on encounters in health care. (In Swedish: Medlidande och empati – reflektionen om möten i hälso- och sjukvården). In: Rehnsfeldt A Arman M (eds) Clinical Caring Science. Caring on a theoretical basis. (In Swedish: *Klinisk vårdvetenskap. Vårdande på teoretisk grund*). Liber, 2020, pp. 102–131.

[44] Graneheim U Lundman B. Qualitative content analysis in nursing research: concepts, procedures and measures to achieve trustworthiness. Nurse Educ Today 2004; 24(2): 105–112. DOI: 10.1016/j.nedt.2003.10.001. 14769454

[45] World Medical Association. WMA Declaration of Helsinki – Ethical principles for medical research involving human subjects. https://www.wma.net/policies-post/wma-declaration-of-helsinki-ethical-principles-for-medical-research-involving-human-subjects/ (2013). 10.1001/jama.2013.281053 24141714

[46] Finnish National Board on Research Integrity, TENK. The ethical principles of research with human participants and ethical review in the human sciences in Finland. https://tenk.fi/sites/default/files/2021-01/Ethical_review_in_human_sciences_2020.pdf (2019).

[47] Hemberg J Bergdahl E Ethical Sensitivity and Perceptiveness in Palliative Home Care through Co-creation. Nursing Ethics 2020; 27(2): 446–460. DOI: 10.77/0969733019849464. 31280654

[48] Lown BA. Toward more compassionate healthcare systems comment on “enabling compassionate healthcare: perils, prospects and perspectives”. Int J Health Policy Manag 2014; 2(4): 199–200. DOI: 10.15171/ijhpm.2014.41. 24847487 PMC4025098

[49] Sinclair S Norris JM McConnell SJ, et al. Compassion: a scoping review of the healthcare literature. BMC Palliat Care 2016; 15(1): 6. DOI: 10.1186/s12904-016-0080-0. 26786417 PMC4717626

[50] Lee Y Seomun G. Role of compassion competence among clinical nurses in professional quality of life. Int Nurs Rev 2016; 63(3): 381–387. DOI: 10.1111/inr.12295. 27353952

[51] Tierney S Seers K Tutton E, et al. Enabling the flow of compassionate care: a grounded theory study. BMC Health Serv Res 2017; 17(1): 174. DOI: 10.1186/s12913-017-2120-8. 28253874 PMC5335833

[52] Bond C Stacey G Field-Richards S., et al. The concept of compassion within UK media-generated discourse: a corpus-informed analysis. J Clin Nurs 2018; 27(15–16): 3081–3090. DOI: 10.1111/jocn.14496. 29700874

[53] Ghafourifard M Zamanzadeh V Valizadeh L, et al. Compassionate nursing care model: results from a grounded theory study. Nurs Ethics 2022; 29: 621–635. DOI: 10.1177/09697330211051005. 35100909

[54] Chen XL Fei Huang F Zhang J, et al. Tertiary hospital nurses’ ethical sensitivity and its influencing factors: a cross-sectional study. Nurs Ethics 2022; 29(1): 104–113. DOI: 10.1177/09697330211005103. 34296649

[55] Weaver K Morse J Mitcham C. Ethical sensitivity in professional practice: concept analysis. J Adv Nurs 2008; 62(5): 607–618. DOI: 10.1111/j.1365-2648.2008.04625.x. 18355227

[56] Durkin J Usher K Jackson D. Embodying compassion: a systematic review of the views of nurses and patients. J Clin Nurs 2019; 28(9–10): 1380–1392. DOI: 10.1111/jocn.14722. 30485579

[57] Gustafsson T Hemberg J Compassion fatigue as bruises in the soul – a qualitative study on nurse. Nursing Ethics 2022; 29(1): 157–170. DOI: 10.1177/09697330211003215. 34282669 PMC8866753

[58] Dekeseredy P Landy CMK Sedney CL. An exploration of work related stressors experienced by rural emergency nurses. Online J Rural Nurs Health Care 2019; 19(2): 2–24. DOI: 10.14574/ojrnhc.v19i1.550.

[59] Finley BA Sheppard KG. Compassion fatigue: exploring early-career oncology nurses’ experiences. Clin J Oncol Nurs 2017; 21(3): E61–E66. DOI: 10.1188/17.CJON.E61-E66. 28524893

[60] Beardsmore E McSherry R. Healthcare workers’ perceptions of organisational culture and the impact on the delivery of compassionate quality care. J Res Nurs 2017; 22(1–2): 42–56. DOI: 10.1177/1744987116685594.

[61] Honkavuo L Lindström UÅ. Nurse leaders’ responsibilities in supporting nurses experiencing difficult situations in clinical nursing. J Nurs Manag 2014; 22(1): 117–126. DOI: 10.1111/j.1365-2834.2012.01468.x. 23409869

[62] Zamanzadeh V Valizadeh L Rahmani A, et al. Factors facilitating nurses to deliver compassionate care: a qualitative study. Scand J Caring Sci 2018; 32(1): 92–97. DOI: 10.1111/scs.12434. 28156018

[63] Salmela S Koskinen C Eriksson K. Nurse leaders as managers of ethically sustainable caring cultures. J Adv Nurs 2017; 73(4): 871–882. DOI: 10.1111/jan.13184. 27732746

[64] Christiansen A O’Brien MR Kirton JA, et al. Delivering compassionate care: the enablers and barriers. Br J Nurs (Mark Allen Publishing) 2015; 24(16): 833–837. DOI: 10.12968/bjon.2015.24.16.833. 26355360

